# No association between waiting time to surgery and mortality for healthier patients with hip fracture: a nationwide Swedish cohort of 59,675 patients

**DOI:** 10.1080/17453674.2020.1754645

**Published:** 2020-04-24

**Authors:** Katarina Greve, Karin Modig, Mats Talbäck, Erzsébet Bartha, Margareta Hedström

**Affiliations:** a Institution of Clinical Sciences, Intervention and Technology (CLINTEC) Karolinska Institutet, Stockholm;; b Perioperative Medicine and Intensive Care, Karolinska University Hospital Huddinge, Stockholm;; c Institute of Environmental Medicine, Unit of Epidemiology, Karolinska Institutet, Stockholm;;; d Department of Orthopaedics, Karolinska University Hospital Huddinge, Stockholm, Sweden

## Abstract

Background and purpose — Waiting time to surgery for patients with hip fractures and its potential association with mortality has been frequently studied with the hypothesis that longer waiting time is associated with adverse outcomes. However, despite numerous studies, there is no consensus regarding which time frames are appropriate, and whether some patients are more vulnerable to waiting than others. We explored the association between waiting time to surgery and short-term mortality and whether sex, age, surgical method, and comorbidity (ASA) modified this association.

Patients and methods — This is a nationwide cohort study of 59,675 patients undergoing hip fracture surgery between January 1, 2013 and December 31, 2017 with a 4-month follow-up of mortality. Data were extracted from the Swedish Registry for Hip Fracture Patients and Treatment (RIKSHÖFT) and mortality was obtained from Statistics Sweden.

Results — Unadjusted analyses revealed an association between waiting more than 24 hours for surgery and increased mortality, primarily for women. However, when stratifying for ASA grade, an association persisted only among patients with ASA 3 and 4. Furthermore, the absolute differences in mortality risk between those waiting less or longer than 24 hours were small. Age, fracture type, and surgical method did not modify the association between waiting time and mortality.

Interpretation — This study suggests that there may be a need for new guidelines, which take into account the heterogeneity of the patient population.

Waiting time to surgery for patients with hip fractures has been studied with the hypothesis that longer waiting time is associated with adverse outcomes for those patients (Ryan et al. 2015, Morrisey et al. 2017, Hongisto et al. 2019). The underlying mechanism as to why prolonged waiting time to surgery would be detrimental is the longer immobilization with a following catabolism (Hedström et al. 2006) as well as the subsequent increased risk of complications. However, there is no consensus regarding what time frames are optimal, and what constitutes a “longer” waiting time varies widely in different studies (Lewis and Wadell 2016).

In Sweden, the latest national guidelines prescribe that all patients should receive surgery within 24 hours (National Board of Health and Welfare 2003). Other countries have similar guidelines: the British National Clinical Guideline Centre (NICE) recommends surgery the same day or the day after hospital admission (NICE 2011). The American Academy of Orthopaedic Surgeons recommends surgery within 48 hours of hospital admission (AAOS 2014). One way to attempt to decrease waiting time to surgery is to institute “fast track care” for patients with hip fracture, often consisting of attempts at early recognition of the hip fracture and thereafter expedient admission to the hospital ward, sometimes bypassing the emergency room entirely (Larsson et al. 2016, Pollmann et al. 2019)

It is not known how, and if, waiting longer than 24 hours for surgery was associated with increased mortality compared with waiting less than 24 hours for surgery for patients with hip fractures in Sweden in recent years. It is further possible that the inconsistent results in previous studies on the risks of adverse outcomes due to prolonged waiting time to surgery may be due to different population characteristics of the study subjects in different studies. Some patient groups may be more vulnerable to waiting than others, which calls for studies looking at subgroups separately.

We explored the association between waiting time to surgery and the 4-month mortality risk in patients with a hip fracture in Sweden between 2013 and 2017, and whether sex, surgical method, age, and comorbidity modified this association.

## Patients and methods

**Figure 1.**
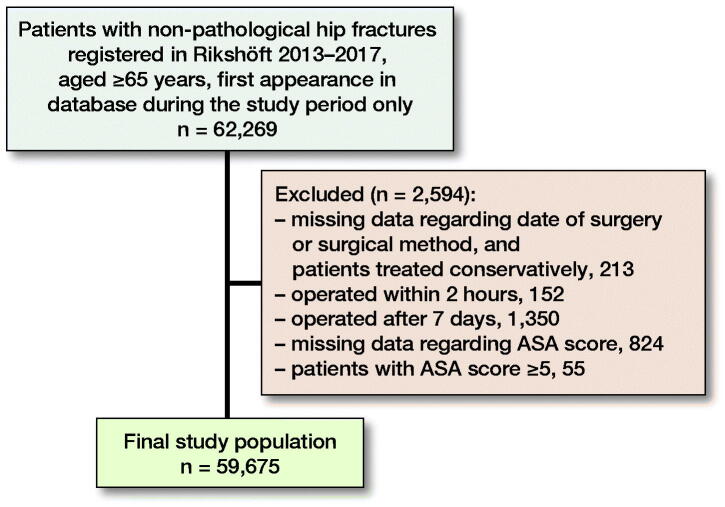
Flowchart of the study population.

This is a nationwide cohort study of patients operated for a hip fracture between January 1, 2013 and December 31, 2017. Patient data were extracted from the Swedish Registry for Hip Fracture Patients and Treatment (RIKSHÖFT), a register with estimated coverage of 80–86% for these years (National Board of Health and Welfare 2014, National Board of Health and Welfare 2018). Exclusion criteria were age < 65 years, pathological fractures (i.e., caused by malignancies, bone cysts, or Paget’s disease), waiting time less than 2 hours (assumed as erroneous reporting) or more than 7 days, ASA score ≥ 5. If an individual was registered more than once during the study period, the first fracture only was considered (Figure 1).

### Variables

The exposure, waiting time to surgery, was measured as the time in hours that elapsed between arrival to hospital and start of surgery as registered in RIKSHÖFT.

The outcome was time to death up to 4 months from the date of surgery. The date of death was obtained from Statistics Sweden (Ludvigsson et al. 2016). Comorbidity was measured through ASA physical status classification system (Dripps 1963). In our material, ASA classification was assessed preoperatively by local anesthesiologists as part of standard preoperative practice, and registered in RIKSHÖFT.

Type of fracture was registered in RIKSHÖFT and adjusted for in the analysis in 2 categories, cervical (consisting of non-displaced, displaced, and basicervical femoral neck fractures) and non-cervical (2-part, multiple-part intertrochanteric and subtrochanteric fractures). Type of surgery was dichotomized into 2 groups: surgical method 1, which included procedures considered more invasive (intramedullary nail, hemiarthroplasty, or total hip replacement); and surgical method 2, less invasive procedures (screw, pin or nail, 2 screws, pins or nails, 3 or more screws, pins or nails, screw, pin or nail with side plate or other).

Living independently before fracture was defined as patients residing in their own homes, with or without assistance from family and/or home care aids.

### Statistics

First, descriptive statistics were produced for the study population. Associations between waiting time and mortality by different fracture types, surgical methods, and ASA score were plotted. Patients with ASA 1 and 2 were compounded into 1 group, and patients with ASA 3 and 4 into another. Next, the proportion of patients who died within 4 months was plotted by hours of waiting time, up to 72 hours. Finally, Cox proportional hazards regression models, using age as the underlying time scale, were used to assess the association between waiting more or less than 24 hours on the time to death, up to 4 months. Crude and adjusted Cox models were performed.

Statistical analyses were conducted using STATA version 14.2 (Stata Corp LLC, College Station, TX, USA).

### Ethics, funding, data sharing plan, potential conflicts of interest

The study was approved by the regional Ethics Committee of Stockholm Dnr 2017/1088-31 and 2018/84-32. The study was funded by the Kamprad Foundation for Entrepreneurship, Research and Charity, reference number 20190135, as well as by grants provided by Region Stockholm (ALF project). This study was based on sensitive individual-level data protected by the Swedish personal data act. Data can therefore only be shared after ethical approval and the consent of the principal investigator. The authors declare no conflicts of interest

## Results

**Figure 2.**
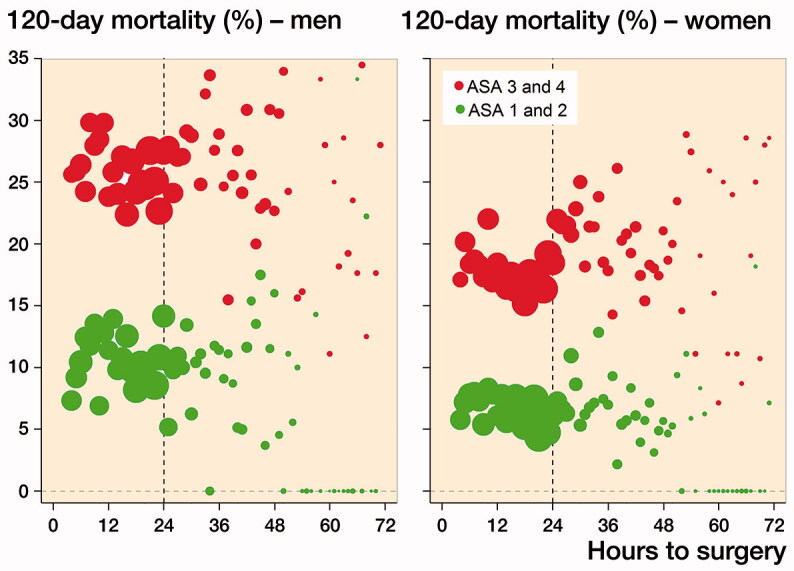
Probability of death within 4 months by waiting time, stratified for ASA score and sex. The size of the dots is relative to the number of patients operated on at each point in time.

Patient characteristics stratified for those undergoing surgery within and after 24 hours, as well as total patient characteristics, are presented in Table 1. 59,675 patients were operated on for a hip fracture and included in the study, of which 68% were women. The mean age was 83 (SD 8) years and the median waiting time to surgery was 20 hours. Overall 30-day mortality was 8%, and 4-month mortality was 16%. A majority of the patients, 70%, underwent surgery within 24 hours. 51% of the patients had a femoral neck fracture. There were no statistically significant differences between the groups who underwent surgery within or after 24 hours with respect to age, sex, fracture type, or surgical method. A larger fraction of the healthier patients (ASA 1 and 2) underwent surgery within 24 hours, compared with the sicker patients (ASA 3 and 4). Overall, a slightly higher fraction of patients were still alive after 4 months in the group that waited less than 24 hours for surgery, 85% compared with 82%. 4-month mortality by waiting time stratified by ASA score and sex is presented in Figure 2.

**Table 1.** Descriptive statistics of study population presented for waiting time to hip fracture surgery less or more than 24 hours (first fractures, 2013–2017). Values are number (%) unless otherwise specified

Factor	≤ 24 hours	> 24 hours	Total
No. of patients	41,569 (70)	18,106 (30)	59,675
Women	28,615 (71)	11,837 (29)	40,452
Men	12,954 (67)	6,269 (33)	19,223
Mean age (SD)	83 (8)	83 (8)	83 (8)
ASA 1	1,845 (76)	571 (24)	2,416
ASA 2	15,278 (73)	5,539 (27)	20,817
ASA 3	21,798 (68)	10,115 (32)	31,913
ASA 4	2,648 (58)	1,881 (42)	4,529
Cervical fractures	20,544 (67)	10,087 (33)	30,631
Non-cervical fractures	21,025 (72)	8,019 (28)	29,044
Surgical method 1 **^a^**	25,501 (69)	11,629 (31)	37,130
Surgical method 2 **^b^**	16,068 (71)	6,477 (29)	22,545
Time until surgery **^c^**	16 (10–20)	32 (27–44)	20 (13–26)
30-day survival **^d^**	93 (92–93)	91 (91–92)	92 (92–93)
4-month survival **^d^**	85 (85–85)	82 (81–82)	84 (83–84)
Living independently before hip fracture	29,468 (70)	12,923 (30)	42,391
On anticoagulants ^e^	4,687 (57)	3,543 (43)	8,230

**^a^** Intramedullary nail, hemiarthroplasty; total hip replacement.

**^b^** Two screws, pins, or nails; screw, pin, or nail with side plate; three or more screws, pins, or nails; others.

**^c^** Median (25th and 75th percentiles) tme in hours. Calculated as time in minutes between arrival at hospital and surgery divided by 60 minutes.

**^d^** Percentage survival with (95% confidence interval)

**^e^** Information concerning anticoagulant use on admission (yes/no) was available for 46,311 patients, i.e., 78% of the study population.

The association between waiting time and death was nonlinear. Men had higher 4-month mortality than women, and patients with ASA 3–4 had higher mortality than patients with ASA 1–2. However, the association between waiting time and mortality was different for the 2 ASA categories and for men vs. women. While the mortality was the same regardless of waiting time for ASA 1–2, there was an initial decline in mortality followed by an increase with longer waiting time for the patients with ASA 3–4, especially among women.

Fully adjusted regression analyses confirmed the stronger association between waiting time to surgery and mortality among women compared with men (Table 2), and that the increased mortality remained only for patients with ASA 3 and 4 when stratifying by ASA (HR 1.1, 95% CI 1.1–1.2) and (HR 1.2, CI 1.1–1.3) (Table 3). Type of fracture and surgical method did not modify the association.

**Table 3.** Adjusted Hazard ratios (aHR) (95% CI) for the association between waiting more than 24 hours compared with surgery within 24 hours and 4-month mortality, stratified by ASA, all patients

	aHR (CI) **^a^**	aHR (CI) **^b^**	aHR (CI) **^c^**
ASA 1	1.17 (0.72–1.89)	1.27 (0.78–2.08)	1.28 (0.78–2.10)
ASA 2	1.05 (0.94–1.17)	1.05 (0.94–1.17)	1.05 (0.94–1.17)
ASA 3	1.13 (1.07–1.19)	1.13 (1.07–1.19)	1.13 (1.07–1.19)
ASA 4	1.17 (1.06–1.29)	1.17 (1.06–1.29)	1.17 (1.07–1.29)

**^a^**Adjusted for age.

**^b^**Adjusted for age and type of fracture.

**^c^**Adjusted for age, type of fracture, and type of surgery.

**Table 2.** Adjusted Hazard ratios (95% CI) for the association between waiting more than 24 hours compared with surgery within 24 hours and 4-month mortality, stratified by sex

	Hazard ratios (95% CI)	
HR adjusted for	Men	Women
Age	1.16 (1.08–1.24)	1.27 (1.20–1.34)
Age and ASA	1.06 (0.99–1.13)	1.15 (1.09–1.22)
Age, ASA, and type of fracture	1.06 (0.99–1.13)	1.16 (1.09–1.22)
Age, ASA, type of fracture, and type of surgery	1.06 (1.00–1.14)	1.16 (1.09–1.22)

When additionally stratifying the analyses for patients younger and older than 85 years we found that the association with waiting more than 24 hours for surgery remained only among the ASA 3 and 4 patients, regardless of age (Table 4).

**Table 4.** Hazard ratios (95% CI) for the association between waiting more than 24 hours compared with surgery within 24 hours and 4-month mortality, stratified by ASA, patients aged 65–85, and patients aged > 85

Group	HR (CI) for waiting time > 24 h	
Age 65–85	ASA 1–2	1.02 (0.85–1.22)
	ASA 3–4	1.11 (1.03–1.19)
Age > 85	ASA 1–2	1.09 (0.96–1.25)
	ASA 3–4	1.22 (1.15–1.30)

Waiting time < 24 h is reference value 1.00

### Sensitivity analysis

For a subset of the patients, 4,850 individuals, there was additional information regarding the time of the fracture (as opposed to arrival time at the hospital). For these patients we re-ran the analyses to see if the association between waiting time and mortality would change. The results were similar to those using time of arrival at hospital.

30-day mortality is an often-used endpoint in other studies. To facilitate comparisons, 30-day mortality was plotted by waiting time and stratified by ASA score and sex, with results similar to when plotting 4-month mortality (Figure 3, see Supplementary data).

## Discussion

Waiting for hip fracture surgery of more than 24 hours was associated with higher risk of death within 4 months but only for patients with ASA score 3 and 4, and primarily for women. Overall the associations between waiting time to surgery and mortality were rather weak, an absolute difference of a couple of percentage points, and OR equal to 1.6 to 1.2. Fracture type and surgical method did not affect the association between waiting time and mortality.

Women in the ASA 3–4 category who underwent early surgery (within 4–10 hours) demonstrated an increased 4-month mortality compared with those undergoing surgery slightly later. This could conceivably be attributed to 2 factors: these women could be the most vulnerable patients and either could have benefited from more careful preoperative optimization, or they were selected for early surgery based on the presumed benefit of expedient surgical intervention. Notably, if the first hypothesis is true, the 24-hour “rule” may lead to inappropriately rushed perioperative management of the sickest women with hip fracture.

There are no comprehensive data regarding reasons for “delay” of surgery in RIKSHÖFT. It is possible that the risk of mortality can differ depending on whether the delay was a consequence of medically related vs. administrative reasons. A previous study from Sweden, however, suggests that waiting time > 36 hours to surgery was detrimental to patients, at least for functional outcome, regardless of reason (Al-Ani et al. 2008). In the same study, administrative reasons were cited in two-thirds of the cases where the patients had waited more than 24 hours for surgery. One potential medical reason for delaying surgery is the need for reversal of anticoagulant medication. However, the proportion of the group with waiting time to surgery within 24 hours who were on anticoagulant medication on hospital admission was 57%, compared with 43% in the group with longer waiting time, which makes it unlikely that treatment with anticoagulants was an important reason for delayed surgery in our material.

The chosen outcome of this study, mortality, does not capture all consequences of the 24-hour rule. There may be benefits such as lower numbers of complications, but also drawbacks such as rescheduled or postponed surgery for patients not suffering from hip fracture. Considering this, other patient-oriented outcome measures need to be studied.

### Comparison with previous studies

What sets our material most apart from many other comparable studies is that waiting time to surgery overall was very short for the entire cohort, which means that the hazards of prolonged waiting time to surgery could be underestimated if compared directly with cohorts with longer waiting times. The heterogeneity of outcome criteria (in-hospital, 30-day, or 4-month mortality) of measures of comorbidities and of clinical settings makes comparisons difficult between our study and previous studies. Overall, our finding of a weak association between increased risk of death and longer waiting time is in line with previous reports (Pincus et al. 2017, Hongisto et al. 2019, Öztürk et al. 2019). However, there are studies that fail to confirm an association between waiting time to surgery and mortality (Majumdar et al. 2006).

Consistent with several previous studies (Endo et al. 2005, Uzoigwe et al. 2013), men in our study had higher overall mortality rate than women. However, there was a stronger association for women in ASA 3–4 (compared with men) between waiting time to surgery and increased mortality, and this has not been previously reported to our knowledge.

Contrary to our results, a Danish study (Öztürk et al. 2019), conducted in a clinical setting similar to ours, found a weak association between waiting time and 30-day mortality in “healthier” patients. This could be attributed to using different measures of comorbidity. The Öztürk study used the Charlson comorbidity index (CCI), while we used the ASA classification.

### Strengths and limitations

This is, to our knowledge, the first study to explore the association between waiting time to hip fracture surgery and subsequent mortality in a nationwide study from a data source with high coverage and validity. In the subgroup analyses, potential confounders were considered and adjusted for whenever possible.

In observational studies, it is not possible to conclude causal relationships between variables. It is possible that factors we consider confounders could really be mediators; this is a limitation that is difficult for us to completely avoid. On the other hand, we know that waiting time to surgery is often affected by system-related factors, which makes it less likely that waiting time to surgery should be affected by for example ASA score or fracture type.

## Conclusion

For patients with an ASA score of 3 or 4, there was a small increase in the risk of 4-month mortality for those who waited at least 24 hours for surgery. The association was stronger for women than for men, and for patients > 85. Fracture type and type of surgery had no impact on the association. Our findings give no support for the hypothesis that surgery within 24 hours reduces mortality risks of hip fracture patients with an ASA score of 1 or 2. Given these differences between men and women, and for patients with different ASA scores, our results suggest that a strict time limit applying to all patients may not be the best strategy.

## Supplementary Material

Supplemental Material

## Supplementary data

Figure 3 is available as supplementary data in the online version of this article, http://dx.doi.org/10.1080/17453674.2020. 1754645
