# Supplementary material for: No association between waiting time to surgery and mortality for healthier patients with hip fracture: a nationwide Swedish cohort of 59,675 patients
Source: Acta Orthop. 2020 Apr 24;91(4):396–400. doi: 10.1080/17453674.2020.1754645 (PMC8023952; doi:10.1080/17453674.2020.1754645)
Supplement: Supplemental Material [file IORT_A_1754645_SM6324.pdf]

Supplementary data

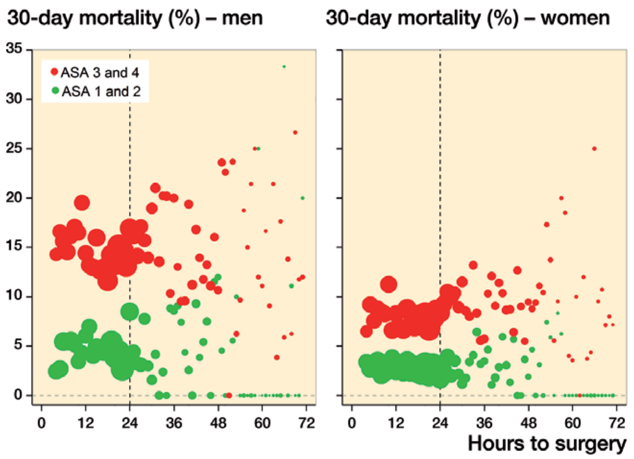

Figure 3. Probability of death within 30 days by waiting time, stratified for ASA score and sex. The size of the dots is relative to the number of patients operated on at each point in time.
